# Application of a forecasting model to mitigate the consequences of unexpected RSV surge: Experience from the post-COVID-19 2021/22 winter season in a major metropolitan centre, Lyon, France

**DOI:** 10.7189/jogh.13.04007

**Published:** 2023-02-03

**Authors:** Jean-Sebastien Casalegno, Samantha Bents, John Paget, Yves Gillet, Dominique Ploin, Etienne Javouhey, Bruno Lina, Florence Morfin, Bryan T Grenfell, Rachel E Baker, VRS study group in Lyon, Elsa Masson, Emilie Bard, Antoine Ouziel, Luc Panetta, Come Horvat, Olivier Claris, Aurélie Portefaix, Marine Butin, Pascal Gaucherand, Jerome Massardier, Mona Massoud, Sandrine Couray-Targe, Anne-Florence Myard Dury, Philippe Vanhems, Sylvie Bin, Stephanie Polazzi, Antoine Duclos, Mehdi Benchaib, Regine Cartier, Marine Jourdain, Martine Valette, Michelle Ottmann, Sylvie Fiorini, Nathalie Rivat, Alexandre Gaymard, Yahia Mekki, Julie Haesebaert, Olivier Terrier

**Affiliations:** 1 Hospices Civils de Lyon, Lyon, France; 2 Centre International de Recherche en Infectiologie (CIRI), Lyon, France; 3 Université Claude Bernard Lyon 1, Lyon, France; 4 Princeton University, Princeton, New Jersey, USA; 5 Netherlands Institute for Health Services Research (Nivel), Utrecht, the Netherlands; 6 Brown University, Providence, Rhode Island, USA

## Abstract

**Background:**

The emergence of COVID-19 triggered the massive implementation of non-pharmaceutical interventions (NPI) which impacted the circulation of respiratory syncytial virus (RSV) during the 2020/2021 season.

**Methods:**

A time-series susceptible-infected-recovered (TSIR) model was used early September 2021 to forecast the implications of this disruption on the future 2021/2022 RSV epidemic in Lyon urban population.

**Results:**

When compared to observed hospital-confirmed cases, the model successfully captured the early start, peak timing, and end of the 2021/2022 RSV epidemic. These simulations, added to other streams of surveillance data, shared and discussed among the local field experts were of great value to mitigate the consequences of this atypical RSV outbreak on our hospital paediatric department.

**Conclusions:**

TSIR model, fitted to local hospital data covering large urban areas, can produce plausible post-COVID-19 RSV simulations. Collaborations between modellers and hospital management (who are both model users and data providers) should be encouraged in order to validate the use of dynamical models to timely allocate hospital resources to the future RSV epidemics.

The emergence of COVID-19 triggered the massive implementation of non-pharmaceutical interventions (NPI) which impacted the circulation of respiratory syncytial virus (RSV) [1]. Whilst RSV activity was disrupted in the first winter, out-of-season RSV resurgence was observed with great heterogeneity across Europe with delays ranging from four to 14 weeks [2,3]. These disruptions may have unexpected consequences on the timing, age of cases, and severity of the RSV outbreaks in the coming years [4]. Anticipating future RSV epidemics is essential for the timely adaptation and preparation of health care resources.

Simple mathematical models have been used to predict pre-COVID-19 incidence patterns of childhood infections in large metropolitan centres [5]. The time-series susceptible-infected-recovered (TSIR) model used herein was previously applied on laboratory surveillance data in the US. It identified climate and school term as typical drivers of pre-COVID-19 RSV dynamics [6] and relaxation of NPI and increasing population susceptibility as the main drivers for post-COVID-19 RSV resurgence [4].

In the Lyon metropolitan centre (1.5 million inhabitants), the 2020/2021 RSV epidemic started with a four-month delay, was smaller than previous winters, and was followed by an unusual RSV circulation until early September 2021 [7,8].

The TSIR model [9] was used to forecast the implications of this delay on future RSV activity in Lyon urban population from September 2021 (week 36/2021) onwards, in order to timely allocate hospital resources to the 2021/2022 RSV epidemic.

## METHODS

The TSIR model was fitted to the demographic data of Lyon and the number of RSV positive tests from week 1/2010 to week 36/2021 to estimate the weekly seasonal transmission rates. Forward simulations were then generated, using the previous estimate parameters fitted to the 2021 demographic data of Lyon and including the different NPI control periods as previously described [4,6]. The model was fitted by minimizing the mean squared errors between predictions and observations (number of RSV positive tests from week 36/2020 to week 36/2021), after accounting for the reporting rate. Using this process, we obtained a 50%, 40%, 30%, and 10% reduction in transmission during the lockdown, school holidays, lockdown with open schools, and curfew periods, respectively. For more details regarding model validation, see Modelling approach section in the **Online Supplementary Document**.

Statistical analyses were performed using R software version 4.1.0 with the following packages (ggplot2; TsiR).

## RESULTS

**Figure 1:**
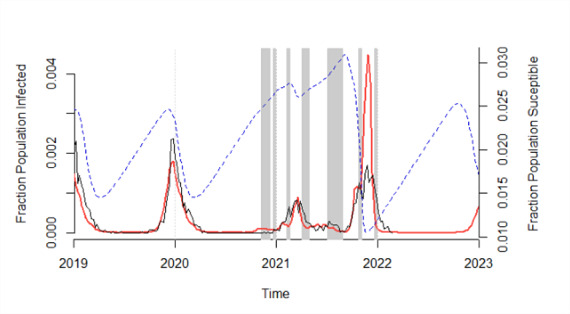
Respiratory syncytial virus (RSV) simulation (red lines) produced on week 37/2021 (before the 20/21 RSV epidemic) compared with the observed RSV laboratory-confirmed cases (dark lines) from week 1/2019 to week 6/2022 (including the 20/21 RSV epidemic). RSV simulation was generated, using the time-series susceptible-infected-recovered (TSIR) model fitted to the 2021 demographic data of Lyon and including the different non-pharmaceutical interventions (NPI) control periods (gray bar). The estimated weekly population proportion susceptible is shown aside (blue dashed lines).

Compared to the pre-COVID-19 outbreaks, the simulation was characterized by an increase in the peak incidence and an earlier timing of this peak at week 48/2021 (usually week 52). The simulation in week 36/2021 was used to inform the paediatric department and a close monitoring of the RSV situation was initiated. The RSV epidemic was soon declared on week 41/2021, closely matching the simulation (**Figure 1**). More resources and staff were gradually allocated to the paediatric hospital to mitigate the fast increase in RSV-related hospitalizations.

A hospitalization peak was then unexpectedly reached in week 44/2021 (week 2 of the autumn holidays) (**Figure 1**). We therefore ran a second simulation in week 44/2022 to adjust for the observed autumn holiday effect (45% rather than 40% reduced transmission). It showed a new peak of higher incidence at week 48/2021 (**Figure 1**). The paediatric department was informed of the second peak timing and allocated resources were maintained until the end of the epidemic on week 3/2022.

## DISCUSSION

When compared to hospital-confirmed cases, the model successfully captured the early start, peak timing, and end of the 2021/2022 RSV epidemic. This information was critical to increase readiness regarding the weekly RSV surveillance data.

In the study context, although there are clear advantages of this simple TSIR model such as the existing literature on its use [4,6], the availability of RSV case time-series (weekly aggregated cases without age structure), and its ease of use (R package), some limitations remain. Since age is not taken into account in the model, it cannot adjust for this factor. This limitation may explain why the number of cases appeared to be overestimated, as the children born during the COVID-19 pandemic contracted RSV at an older age, thus reducing the risk of hospitalization [7]. Second, the model does not take into account the possibility of re-infection. Still, the model is able to broadly capture the impact of COVID-19 NPI on RSV transmission and accurately predict the timing of future outbreaks. The specificity of the TSIR model used at the metropolitan level may explain how this simple deterministic model is able to capture the dynamics of such a complex epidemic [5].

The increase in the peak incidence was caused, in the simulation, by an increase in the proportion susceptible children in the population, due to a softened 2020/2021 RSV epidemic (**Figure 1**). The early start was caused by an increased level of RSV circulation (**Figure 1**) and the seasonal transmission at week 37/2021. These results support the hypothesis that a relaxation of NPIs, school holidays, seasonal variation of transmission, and increased population susceptibility are currently the main drivers of RSV dynamics [10].

## CONCLUSIONS

The current global RSV situation emphasizes the need for plausible simulations that can be combined with the different streams of surveillance data and field expertise to timely allocate adequate local hospital resources to RSV epidemics. Further research is needed to define good modelling practices for the hospital setting. This represents an opportunity to strengthen the collaboration between modellers and hospital clinical management, who are both data providers and model users. Healthcare professionals could benefit from open access, easy-to-use, forecasting models that produce plausible RSV simulations with limited data set requirements, while modellers could benefit from direct access to comprehensive databases and the expertise of health care workers.

## Additional material


Online Supplementary Document
