# Supplementary material for: Application of a forecasting model to mitigate the consequences of unexpected RSV surge: Experience from the post-COVID-19 2021/22 winter season in a major metropolitan centre, Lyon, France
Source: J Glob Health. 2023 Feb 3;13:04007. doi: 10.7189/jogh.13.04007 (PMC9893715; doi:10.7189/jogh.13.04007)
Supplement: Online Supplementary Document [file jogh-13-04007-s001.pdf]

ONLINE SUPPLEMENTARY DOCUMENT

Title: Application of a forecasting model to mitigate the consequences of unexpected RSV surge: experience from the post-COVID-19 2021/22 winter season in a major metropolitan center, Lyon, France

Authors: Jean-sebastien Casalegno1,2,3(http://orcid.org/0000-0003-3271-9856), Samantha Bents4, John Paget 5,Yves Gillet, Dominique Ploin (https://orcid.org/0000-0002-9668-7606), Etienne Javouhey1,3 (https://orcid.org/0000-0002-6480-0758), Bruno Lina1,2,3 (https://orcid.org/0000-0002-8959-2123), Florence Morfin1,2,3 (https://orcid.org/0000-0002-1962-808X), VRS study group in Lyon, Bryan T Grenfell1,2,3 (http://orcid.org/0000-0003-3227-5909), Rachel E Baker1,2,3 (http://orcid.org/0000-0002-2661-8103).

Data Source description

The RSV dataset was obtained from the Lyon hospital laboratory database. All respiratory samples (nasopharyngeal aspirates, nasal/throat/oral swabs, tracheobronchial aspirates, and broncho-alveolar lavages) taken from patients with respiratory tract infection (upper or lower/mild or severe) and received by the virology laboratories from in- and outpatients were tested for RSV and included in our analysis. The majority of cases were younger than 1 year old (60%) and older than 65 years old (20%), as previously described (3). The total number of RSV cases per season varies between 442 (2020/2021 RSV season) and 999 (2018/2019).

The epidemiological reports are available on the Lyon University Medical Hospital website: https://www.chu-lyon.fr ; and upon request at ghn.bulletinbehcl@chu-lyon.fr

Population data for Lyon were obtained from publicly available combined files via the open access INSEE database (https://www.insee.fr/fr/information/2410988).

Data regarding NPI periods were obtained from publicly available information websites. Given the importance of school opening (Li You *et al* 2022), total lockdown (with schools closed) and partial lockdown (without school closure) were considered separately.

Variable	Value	Source
Population size :	2010 : 1293164<br>2011: 1306972<br>2012: 1324637<br>2013: 1336994<br>2014: 1354476<br>2015: 1370678<br>2016: 1381349<br>2017: 1385927<br>2018: 1399192<br>2019: 1411571<br>2020: 1411571<br>2021: 1411571	https://www.insee.fr
Weekly Birth rate	2011: 408<br>2012: 408<br>2013: 408<br>2014: 408<br>2015: 409	https://www.insee.fr

	2016: 401<br>2017: 397<br>2018: 393<br>2019: 386<br>2020: 347<br>2021: 347	
NPI periods	Week 45 /2020 (length 5 weeks): Partial Lockdown<br>Week 51/ 2020 (length 2 weeks): Total Lockdown<br>Week 6/ 2021 (length 2 weeks): School<br>Week 14/2021 (length 3 weeks): Total Lockdown<br>Week 17/2021 (length 1 weeks): Partial Lockdown<br>Week 27/2021 (length 8 weeks): School<br>Week 43/2021 (length 2 weeks): School<br>Week 51/2021 (length 2 weeks): School	https://www.wikipedia.fr<br>COVID-19 Government Response Tracker, 2020<br>https://covidtracker.bsg.ox.ac.uk/

Modeling approach

Our modeling approach follows two steps.

We first used the time-series TSIR model, a discrete time adaptation of the SIR model, to estimate the bi-weekly transmission rate β_t that captures the seasonal trend in transmission.

The model parameters were as follows: α = 0.97; mean βt = 4.83e-05

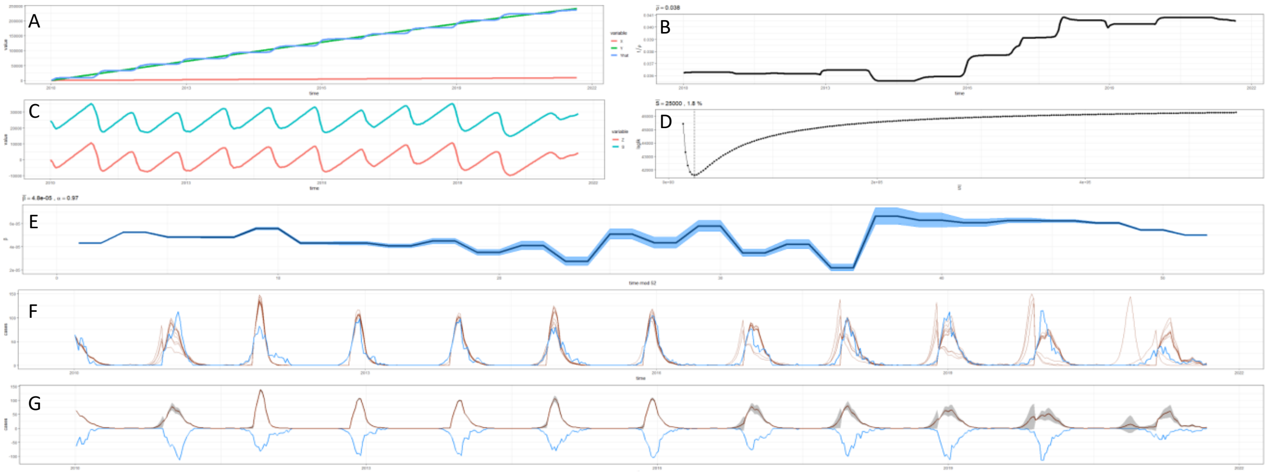

Figure S1. Output results from the runtsir function for Lyon (Week 1/ 2010-Week 37/ 2020). A) and B) are the cumulative births against cumulative cases regression and estimated reporting rate C) and D) are the reconstructed Susceptible Fraction of the population and profiled mean number of susceptible individuals across the time-series, E) is the bi-weekly transmission rate βt with the fixed α and mean β and F) and G) are the data (blue) against 10 randomly chosen stochastic simulations (red) and the (inverse) data against mean of the simulations with confidence intervals (2).

Second, we generated forward simulations using seasonal transmission rates, βt, assuming a constant annual population and birth rate (based on average population and average birth rates from the 2020 time-series). Model results are shown in terms of incidence per capita. The simulations were initially run for 40 years to remove transient dynamics and the control period was introduced to the model by lowering the seasonal transmission rates by a fixed proportion, starting on week 45/2020 (the week when the first lock-down within the seasonal RSV circulation period started).

The beta reduction values of the model were fitted by minimizing the mean squared errors between predictions and observations from week 36/2020 to week 36/2021 (period of the atypical 2020/2021 delayed epidemic), after accounting for the reporting rate. Using this process, we obtained a 50%, 40%, 30%, and 10% reduction in transmission during the total lockdown, school holidays, partial lockdown (with schools opened), and curfew periods, respectively.

For all models, α, the constant that captures heterogeneities in mixing and the discretization of a continuous time process, was fixed at 0.97 as previously reported (1,4).

<u>Simulation 1</u>

Simulation 1, performed on week 36/2021, estimated the main RSV peak on week 48/2022

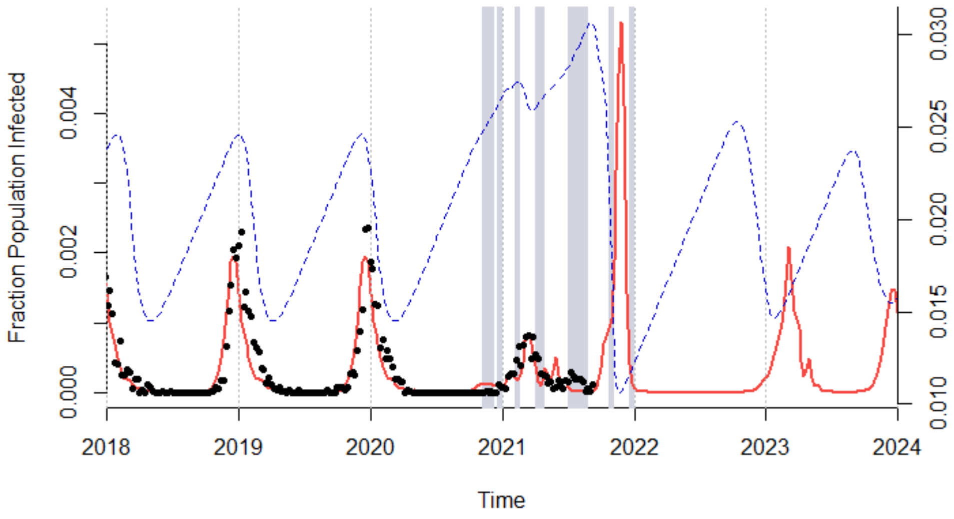

<u>Figure S2:</u> RSV simulation (red lines) produced on week-37/2021 (before the 20/21 RSV epidemic) compared with the observed RSV laboratory-confirmed cases (dark lines) from week-1/2019 to week-36/2021 (including the 20/21 RSV epidemic). RSV simulation was generated, using the TSIR model fitted to the 2021 demographic data of Lyon and including the different NPI control periods (gray bar). The estimated weekly population proportion susceptible is shown aside (blue dashed lines).

<u>Simulation 2</u>

Simulation 2, performed on week-44/2022 to adjust for the observed autumn holiday effect (45% rather than 40% reduced transmission), also estimated the main RSV peak on week 48/2022 but with a lower incidence.

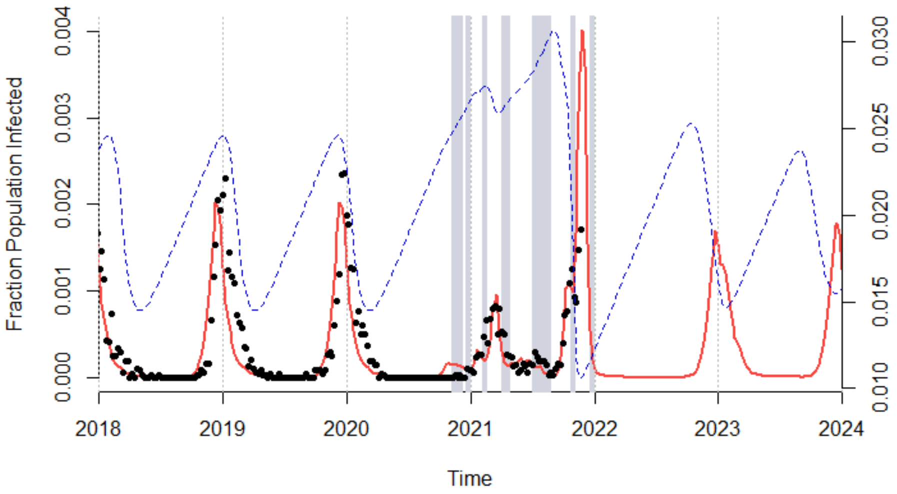

Figure S3: RSV simulation (red lines) produced on week-44/2021 (before the 20/21 RSV epidemic) compared with the observed RSV laboratory-confirmed cases (dark lines) from week-1/2019 to week-43/2021 (including the 20/21 RSV epidemic). RSV simulation was generated, using the TSIR model fitted to the 2021 demographic data of Lyon and including the different NPI control periods (gray bar). The estimated weekly population proportion susceptible is shown aside (blue dashed lines).

Simulation accuracy prediction

To assess the short-term prediction accuracy we compared the timing and overall size of the outbreak to what was predicted with the observed hospitalizations/week.

	Observed	Simulation 1	Simulation 2
Main Peak Timing	Week-48/2021	Week-48/2021	Week-48/2021
Main Peak Incidence	2184	4372	3725
Mean squared errors between Incidence predicted and observed from Week-37/2021 to Week 10/2022	NA	40 229 510,55	36 991 604,79

References:

1. R. E. Baker et al., Epidemic dynamics of respiratory syncytial virus in current and future climates. Nat. Commun. 10, 5512 (2019).
2. Becker AD, Grenfell BT. tsiR: An R package for time-series Susceptible-Infected-Recovered models of epidemics. PLoS One. 2017 Sep 28;12(9):e0185528. doi: 10.1371/journal.pone.0185528. PMID: 28957408; PMCID: PMC5619791.
3. Casalegno JS, Ploin D, Cantais A, et al. Characteristics of the delayed respiratory syncytial virus epidemic, 2020/2021, Rhône Loire, France. Euro Surveill. 2021;26(29):2100630. doi:10.2807/1560-7917.ES.2021.26.29.2100630
4. K. Glass, Y. Xia, B. T. Grenfell, Interpreting time-series analyses for continuous-time biological models—Measles as a case study. J. Theor. Biol. 223, 19–25 (2003).

5. Li Y, Wang X, Cong B, Deng S, Feikin DR, Nair H. Understanding the Potential Drivers for Respiratory Syncytial Virus Rebound During the Coronavirus Disease 2019 Pandemic. J Infect Dis. 2022 Mar 15;225(6):957-964. doi: 10.1093/infdis/jiab606. PMID: 35030633; PMCID: PMC8807230.
